# Interaction of culture and grief amongst women who terminated a pregnancy in adolescence: A narrative approach

**DOI:** 10.4102/curationis.v44i1.2247

**Published:** 2021-11-23

**Authors:** Botshelo R. Sebola

**Affiliations:** 1 Department of Health Studies, School of Social Sciences, University of South Africa, Pretoria, South Africa

**Keywords:** adolescence, approach, culture, grief, interaction, narrative approach, terminated pregnancy, women

## Abstract

**Background:**

Culture plays a vital role in resolving grief in African communities. However, women who terminate a pregnancy in adolescence are typically not exposed to cultural rituals that could ease their grief.

**Objectives:**

The purpose of this article is to explore the interaction of culture and grief amongst women who terminated a pregnancy in adolescence.

**Method:**

A qualitative exploratory study was undertaken using a narrative approach. Unstructured interviews were conducted to solicit narratives from 11 women who terminated a pregnancy in adolescence.

**Results:**

Data were analysed through narrative, thematic data analysis. Three themes emerged from the findings: delayed post-traumatic growth, low body esteem and an alteration in the development of maternal identity.

**Conclusion:**

The study intended to explore the interaction of culture and grief amongst women who terminated a pregnancy in adolescence. The researcher determined that women who had not honoured their culture because of the secrecy surrounding the termination of pregnancy had delayed healing and an altered self-image.

## Introduction

Culture plays a vital role in resolving grief in African communities. Africans are rooted in their cultural beliefs, and tradition permeates almost every aspect of societal rituals, including death and burials (King 2013). Death rituals have played an important role in helping individuals cope with death and celebrate the death of a loved one (Obare 2019). The overall purpose of death rituals is to support those who remain and offer acceptance and a celebration of life for those who have lost a loved one.

As many Africans believe that death, including the death of a foetus (by any means), may result in misfortune if the deceased did not receive a dignified send-off, African cultures enforce the practice of formal death ceremonies. These ceremonies typically involve guests, the slaughtering of an animal, food and prayer in celebrating the life and death of the deceased (Lagat 2018). How death is viewed and celebrated will vary amongst religions and cultures. Death rituals are believed to let the deceased rest in peace and allow the healing of those who are close to the deceased. The Bukusu people of Kenya practice a ritual of naming family members after the deceased, believing that the dead live amongst them and will bless them (Obare 2019). These practices are meaningful and assist the bereaved in healing emotionally. Hoy (2013), in his findings from investigating funeral practices, established five common components of death rituals amongst different cultures, namely: (1) significant symbols, (2) gathered community, (3) ritual action, (4) connection to heritage and (5) transition of the corpse. The significant symbols are used to honour the deceased, the gathered community provides support to the bereaved, the ritual action encourages mourners to be active, connection to heritage symbolises the value of cultural practices and customs and the corpse’s presence is normally used in a ritualistic manner (Hoy 2013). A common generalisation is that the execution of these rituals is essential in maintaining harmony and balance between the physical and the spiritual world (Baloyi & Makobe-Rabothata 2014).

The World Health Organization (WHO) estimates that close to 21 million adolescent girls aged 15 to 19 years fall pregnant every year and 5.6 million of these pregnancies end in abortion (WHO 2020). The majority of these terminations take place without the knowledge of parents or other support systems. Moreover, adolescents differ from older women undergoing the termination of a pregnancy in several ways. In addition to being intellectually, morally and emotionally less mature, their life experiences and cultural knowledge are limited (Coleman et al. 2007).

## Problem statement

Women who terminated a pregnancy in adolescence are seldom exposed to cultural rituals related to death that could afford them a reprieve from their grief. The stigma related to the termination of pregnancy causes many of these women not to openly acknowledge their loss but rather grieve in silence, thereby forsaking public mourning and subsequent social support (Doka 2009).

Whilst cultural rituals play a significant role in easing grief in African communities, in South Africa, the setting of this study, there is limited research on the interaction of culture and grief amongst women who terminated a pregnancy during adolescence. As most women who terminated a pregnancy in adolescence are not knowledgeable about cultural death rituals that could ease their grief, there is a need to explore the interaction of culture and grief amongst women who terminated a pregnancy in adolescence.

## Purpose

The purpose of this study was to explore the interaction of culture and grief amongst women who terminated a pregnancy in adolescence.

## Objectives

- To describe the role played by culture in the resolution of grief
- To describe how culture and grief interact

## Definition of key concepts

### Adolescence

Adolescence is a phase of life when a person is transitioning from childhood to adulthood. This transformation is marked by the maturity of sexual functioning (Kinghorn et al. 2018; Weiten 2013). Though there are varying views, but adolescence is often understood as the age between 10 and 24 years (Bruce, Lunt & McDonagh 2017; Sawyer et al. 2018; Weiten 2013). This study aligns with the South African view, which defines adolescents as those aged between 10 and 19 years (Health e-News 2015).

### Culture

De Oliveira Cardoso et al. (2020) defined culture as:

[*A*] way in which societies structure the role of rites and rituals. Rite includes a broader category such as the rite of passage, or healing, while the ritual is a set of gestures and actions that make the rites. (p. 2)

The researcher used this definition to apply the context of culture in this article. Within each culture there is a mix of death rituals, beliefs about death and traditions related to burial as well as expectations about maternal identity and the woman’s protection of her pregnancy (Souza & Souza 2019). For women who are grieving the termination of a pregnancy, these African cultural practices, if followed, could help promote a feeling of emotional stability, security and emotional healing.

### Grief

Grief has specific social and cultural meanings. Delahanty and Washburn (2014) explained grief as a universal emotional experience that most people go through following the loss or death of a loved one. However, there are a variety of individual differences concerning the intensity, duration and ways of expressing this emotion (Genevro, Marshal l & Miller 2004). Feelings of anger, sadness, social and intellectual difficulties are typical of the experience of loss. With time comes acknowledgement and acceptance, and these emotions are normally resolved; gradually, the grieving person is able to reflect and normalise their situation (Genevro et al. 2004; Swan et al. 2021).

### Interaction

According to Wang, Elston and Zhu (2011), interaction relates to the transitional action between things; it can be understood as the reciprocal influence between culture and grief. In this study, the interaction between culture and grief means that the cultural practices around death could have a positive or negative impact on those left behind, depending on whether these practices are properly adhered to. African traditional religion requires specific burial rituals to ensure a proper send-off for the departed and avoid potential disasters (Ezenweke 2008; Obasi 2002). Therefore, a woman who terminated a pregnancy but failed to provide an appropriate send-off for the foetus may be haunted by the fear of impending calamities.

## Research methods and design

A qualitative, exploratory, narrative methodology (Trahar 2009) was followed to explore the stories of 11 women who terminated a pregnancy in adolescence and their experiences of the interaction of culture and grief. The study was conducted in Gauteng, South Africa. The researcher’s contact details were included in the consent form, and the participants were asked to contact the researcher or the Chair of the Review Board should they have any questions regarding the study.

### Sampling and participants

Purposive and snowball sampling techniques were used to recruit 11 participants who terminated a pregnancy in adolescence. Women who terminated a pregnancy in adolescence were difficult to recruit because of the stigma attached to this phenomenon in most African societies (Loi et al. 2018). Participants were recruited through a community health centre that works with women who want to terminate a pregnancy. The centre’s management distributed flyers to potential participants, containing an invitation to be part of the study, the purpose of the study, expectations of participants, benefits of the study, an overview of participants’ rights and confidentiality and the Chair of the Review Board and researcher’s contact details. Through this sampling method, five potential participants contacted the researcher and were willing to participate in the study. The snowball technique was then used to recruit an additional six participants (Berg 2001; Cohen & Arieli 2011). All the participants had to meet pre-determined inclusion criteria, namely (1) participants had to be women aged between 20 and 50 years, (2) residing in Gauteng province of South Africa and (3) had terminated a pregnancy in adolescence.

### Informed consent

A thorough explanation about the study was given to the participants who called and expressed an interest in taking part. Participants were asked to sign a consent form after they were informed about the study’s intent and how the findings would be used. The consent form also stipulated that participants should not use their names but pseudonyms to ensure their confidentiality. The consent form was signed at a place and time agreed upon by both the researcher and the participant. After signing the consent form, an interview appointment was scheduled and participants were asked not to use their airtime to call the researcher but to alert her of their availability so that she could contact them. The issue of voluntarism was emphasised throughout the study and participants were free to withdraw from the study at any time.

### Data collection

Data were collected from July 2017 to December 2017. Unstructured audio-recorded interviews were conducted to solicit data from 11 women who terminated a pregnancy in adolescence. Participants gave their permission for the interviews to be recorded. In accordance with the narrative approach to data collection, the interviews started with a broad opening statement: ‘Tell me how culture has interacted with your grief after you terminated a pregnancy in adolescence’. Participants freely shared how their failure to engage in cultural rituals affected them and explained the connection between culture, grief and moving on with their lives. A set of probing questions were prepared, but they were ultimately not required because the participants gave a detailed account of their experiences during the interviews. Such questions included: (1) How has your failure to engage in cultural rituals affected you? (2) How do you feel after terminating a pregnancy? and (3) Does culture and grief, and moving on with your life, have any connection?

### Data analysis

The interviews were transcribed verbatim, and the researcher read them several times to gain an understanding of the participants’ experiences (Riessman 2008). The interviews were envisioned as units of analysis. Sentences were viewed as units of meaning that were later compressed according to their content and codes; the codes were furthermore arranged into categories and subcategories based on similarities. Ultimately, themes were formulated from these categories. A sample of the transcripts was then given to participants to confirm whether their views had been captured clearly, and they all confirmed that the transcript was a true reflection of their experiences (Kaewkungwal & Adams 2019).

## Findings of the study

The participants were aged between 20 and 42 years (see Table 1). Six participants had terminated a pregnancy after being coerced by their parents or partners; they were unwilling to terminate but believed they did not have a choice at the time. Four participants terminated a pregnancy out of fear of being found out and one participant terminated pregnancy as a way of getting back at her partner, who had been involved in many relationships. The data analysis revealed three themes: delayed post-traumatic growth, low body esteem and an alteration in the development of a maternal identity.

**TABLE 1:** Biographic characteristics of participants.

Biographic characteristics	Frequency (*N* = 11)
**Age**	
20–24 years	4
25–29 years	2
30–34 years	2
35–50 years	3
**Level of education**	
Primary education	2
Secondary education	6
Tertiary education	3
**Ethnicity**	
Tswana	5
Zulu	3
Pedi	3
**Marital status**	
Single	8
Married	1
Cohabitation	2
**Employment**	
Employed	5
Unemployed	2
Self-employed	4
**Number of terminated pregnancies**	
1	10
3	1
**Age at first termination of pregnancy**	
15 years	2
16 years	2
18 years	3
19 years	4

*Source*: Makutoane, M.E., 2016, ‘Choice on termination of pregnancy: Its impact on the woman’s health’, Unpublished dissertation. University of South Africa, Pretoria.

### Delayed post-traumatic growth

Post-traumatic growth is the ability to attain deep and progressive personal development. This development has an impact on relationships, appreciation for life, growth, functioning and self-perception as a strong person following a traumatic event such as terminating a pregnancy or living in poverty, amongst others (Tsai et al. 2016). With age and consequent life experiences, people experience more post-traumatic growth (Nuccio & Stripling 2021).

All the participants in the study experienced delayed post-traumatic growth because none of them had fully embraced their circumstances after terminating a pregnancy in adolescence. Participants shared:

‘I still feel guilty when I see children who may have been of my daughter’s age, and I cannot talk to anyone at home or even my partner. I feel it will be better if I had gone through some cleansing ritual to help me adapt.’ (N9, 26-year-old who terminated a pregnancy at 15)

‘I feel such great antipathy towards my boyfriend. He now wants a baby and when I get pregnant, I go behind his back and terminate it. I had already terminated three, I can’t help it. I cannot allow myself to hold a baby in my hands. God will punish me for it.’ (N6, 26-year-old who terminated a pregnancy at 19)

‘My grandmother was a Sangoma (traditional doctor), and I now see her all the time when I have conjugal rights with my husband like she wants to know why I aborted my first child. As my husband does not know what I did in adolescence, I feel so much guilt.’ (N11, 36-year-old who terminated a pregnancy at 16)

### Low body esteem

Low body esteem was another finding of the study and was defined in the way the participants understood their worth in relation to cultural expectations. Body esteem correlates with self-esteem and lower body esteem reflects lower self-esteem (Olenik-Shemesh, Heiman & Keshet 2018). The following narrative quotes support this finding:

‘I feel ugly and inhuman, my ugliness is within me, how can I as a woman live my life as if nothing has happened? The termination of pregnancy has left me with an emptiness inside, all I do is eat and walk around, how I look physically no longer matters to me.’ (N2, 30-year-old woman who terminated a pregnancy at 18)

‘In my culture, there is a saying that “a woman is regarded as a woman if she can protect her babies from conception to adulthood” What did I do? I have failed this simple and well-known idiom in my culture. I terminated my pregnancy when I was 4 months pregnant … It was not performed in the hospital, and it was carried out by my mother, it was a boy … I looked and my mother was wrapping it in a cloth. I can never forget that picture, it makes me feel like a true murderer. Since then I no longer care about myself as a person, I feel I do not deserve anything good in life.’ (N8, 24-year-old woman who terminated a pregnancy at 15)

### Alteration in the development of a maternal identity

An alteration in the development of a maternal identity was another theme that emerged from the analysis. The development of a maternal identity is the process of adopting maternal behaviours (Özkan & Polat 2011). In this study, participants felt they were unfit to be mothers, yet they all experienced the cultural expectation of bearing and raising children. The participants shared the following statements in this regard:

‘How do I even call myself a woman when I could not raise my baby? I look at other women who struggle and still manage to have children and accepted them as God’s gift, why couldn’t I do that.’ (N10, 35-year-old who terminated a pregnancy at 19)

‘I feel less than a woman and my partner also affirms that from the remarks he says about my mothering ability. According to him, I should not have had babies (referring to the two I had after termination). He is probably telling the truth.’ (N5, 32-year-old woman who terminated a pregnancy at 19)

## Discussion

The purpose of this study was to explore the interaction of culture and grief amongst women who terminated a pregnancy in adolescence. This study was designed to gain an understanding of the role played by culture in resolving grief and how culture and grief interact amongst women who terminated a pregnancy in adolescence. Participants’ delayed post-traumatic growth demonstrated how intertwined culture and grief are for women who terminated a pregnancy. The African cultural system believes that the deceased are alive in the spiritual world of the living dead. Consequently, many Africans believe death rituals are meant to ease the deceased’s transition from the world of the living to the world of the living dead. These rituals ensure peaceful relations between the living and the living dead, whereby the living dead remain connected to their families and community members (Baloyi & Makobe-Rabothata 2014; Lagat 2018). It is believed that failure to observe these rituals could result in various calamities befalling the family or individuals (Ezenweke 2008).

All participants did not engage in any rituals, either because they did not know about them or the rituals conflicted with their Christian faith. Obare (2019) determined that some death rituals can creatively and harmoniously be incorporated and performed within Christian churches. Some participants in this study expressed that cultural practices are important because the termination of a pregnancy is a death experience. Different rituals assist the bereaved in accomplishing healing, for instance, informing others of the death and thereby reaching out for support, accepting the parting of a loved one, sharing the pain of grief with family and the community, adjusting to life after the loss and resuming normal functioning as individuals, as family, and as a community (Obare 2019).

Women who terminated their pregnancies carry the burden of hiding their grief from their support system and lack adherence to symbolic behaviours, which according to one’s culture, surpasses family virtues.

Despite the counselling some participants received before and after terminating their pregnancy, they still struggled with the loss. Their interpersonal relationships and their self-worth continued to suffer (Coleman et al. 2007). This is a clear demonstration of their delayed post-traumatic grief.

The second theme that emerged from the findings related to participants’ low body esteem. Body image is the mental representation of the individual’s attitude dimension, which is related to behaviours such as overeating and thoughts and feelings about one’s physical appearance (Cash & Smolak 2011). This statement affirms what the participants reported during their interviews.

Participants understood their value as women in relation to the cultural expectation of protecting their babies from conception to adulthood, a responsibility they failed to uphold. This perception caused them to neglect their physical appearance and had adopted an ‘I do not care how I look’ attitude, which did not help their self-esteem. Body esteem and self-esteem are closely related as indicators of well-being; therefore, a woman with high self-esteem also has high body esteem. According to Maslow’s hierarchy of needs, one must have positive self-esteem to attain a high level of functioning. Women who terminated a pregnancy in adolescence may find that their body esteem has been altered, resulting in a proneness to psychological distress (Olenik-Shemesh et al. 2018).

The final theme that emerged from the data analysis was an alteration in the development of a maternal identity. The development of a maternal identity reflects a transition from feelings associated with pregnancy, which may include hope, distress and fear, the attainment of and coping with a maternal role (Hendricks-Munoz & Mayers 2014). A motherhood identity takes cognisance of the composite interaction between individuals within their socio-cultural context (Abrams & Curran 2011). In Africa, specifically South Africa, a woman is expected to share her maternal experiences with peers and family, ask for assistance and read about maternal expectations to develop a maternal identity (Gross-Spector & Cinamon 2018). In this study, the women had to hide their pregnancy; those who did not hide it were rebuked for being pregnant and were therefore not supported. This lack of culturally expected support altered their maternal identity. Support is rated as the most important facilitator of maternal role development (Kendall-Tackett 2014).

## Conclusion

The purpose of this study was to explore the interaction of culture and grief amongst women who terminated a pregnancy in adolescence. It was determined that culture plays a vital role in participants’ experiences of grief because their failure to observe cultural practices often led to prolonged grieving. A lack of support from significant others before and after terminating a pregnancy altered participants’ development of a maternal identity. Delayed grieving caused participants to neglect their bodies, which was detrimental to their self-esteem and well-being. The study also revealed the lack of knowledge amongst young women about death rituals and their meaning and importance in resolving grief.

This research contributes to the growing body of knowledge that highlights the importance of culture in the many spheres of life, including aspects of grief. It further affirms the notion that distress often occurs because of a clash between two competing concepts, in this context, culture and grief.

## Limitations of the study

The small sample makes it difficult for this study to be generalised. The sample was small because the researcher experienced difficulty accessing participants who were willing to share information on this sensitive topic.

The study was qualitative by nature; however, the themes that emerged might be better measured quantitatively, using questionnaires from existing instruments, such as the maternal alteration sub-scale. The author notes that this study could form the basis of a larger quantitative study that explores these sub-scales.
